# Supplementary material for: Recovery in cognitive motor dissociation after severe brain injury: A cohort study
Source: PLoS One. 2020 Feb 5;15(2):e0228474. doi: 10.1371/journal.pone.0228474 (PMC7001945; doi:10.1371/journal.pone.0228474)
Supplement: Attachment — Submitted filename: Response to Reviewers.docx [file pone.0228474.s002.docx]

# **Author’s responses to the Reviewer’s comments**

**Journal:** PLOS ONE

**Manuscript ID:** PONE-D-19-26389
**Title:** Recovery in cognitive motor dissociation after severe brain injury: A cohort study

**Authors:** Jane Jöhr, Floriana Halimi, Jérôme Pasquier, Alessandro Pincherle, Nicholas Schiff, Karin Diserens.

We appreciate the time and effort of the editor and the referees for reviewing the manuscript. We have addressed all issues raised in the review report and hope that the revised manuscript corresponds to the journal publication requirements.

The modifications are highlighted in **bold** in the marked-up copy of the original version (file labeled 'Revised Manuscript with Track Changes').

**Responses to comments of Reviewer-1**

**COMMENT 1: It would be helpful to have more information about what is meant by the use of video recordings as described in the method section. Is it the case that measurements were taken via video recording and if so were the assessments being administered at the time of the recording, or was this but together ad hoc afterwards? if the latter, some discussion should be made in the limitation section regarding nonstandardized procedure.**

Thank you for raising this methodological issue. Indeed, part of the outcome measurements were collected retrospectively, based on the electronic medical records, interdisciplinary therapeutic reports and video-recordings of each patient at admission and discharge. As part of the unit’s routine guidelines, each patient’s complete neurological status at admission and discharge are documented through video recordings, which allow any trained clinician to score offline standard outcome measures such as those used in the present study. Moreover, many therapeutic sessions (i.e., physical, occupational, neuropsychological and speech therapy) were also routinely video-recorded to prevent any doubt on review for the functional and cognitive scoring procedure. A trained neuropsychologist reviewed all well-documented retrospective data; hence, we expect any potential error to be kept to a minimum.

This methodological limitation is now addressed: details have been added to the Methods section and are discussed in the limitations part of the Discussion section.

**COMMENT 2: It is unclear whether results may be effected by variability in the trajectory of recovery for the non-traumatic brain injury group, given that this group is comprised of ischemic, anoxic, toxic, and tumor-related brain injuries. It may be better to limit analyses instead to TBI versus hemorrhagic patients. Alternatively, the potential for different effects in these subgroups should be analyzed, more clearly stated, and/or considered in the conclusion section.**

Thank you for this suggestion concerning clarity of results. The main objective in the present study is to determine the effect of the diagnosis (CMD, DOC and Non-DOC) and not the effect of etiology on the response variables. However, if the etiology has an impact on the latter, it must be considered in multivariable analyzes. Originally, we did this by introducing a variable that distinguishes TBI patients from Non-TBI patients in the regression models. We did not observe any effect of etiology on the differences between patients groups. On your recommendation, we recalculated the outcomes by including a more distinct etiology variable in the Non-TBI group (Hemorrhagic, Ischemic, Anoxic, Toxic, Tumoral). The results were unchanged and we did not observe any effect on the adjusted differences in Table 2.

A sentence regarding the absence of effect of etiology on the differences between groups has been added to the Results section and data are not shown, given the unchanged results.
